# Reliability and validity of a simplified touch experiences and attitudes questionnaire for Chinese college students

**DOI:** 10.1371/journal.pone.0295812

**Published:** 2024-01-02

**Authors:** Lei Yang, Xuelian Li, Zhijie Xie, Lujun Shen

**Affiliations:** 1 Faculty of Psychology, Tianjin Normal University, Tianjin, China; 2 School of Psychology, Xinxiang Medical University, Xinxiang City, Henan Province, China; 3 School of Education, Hebei University, Baoding City, Hebei Province, China; Southwest University, CHINA

## Abstract

Touch cultures have both differences and commonalities in different regions. The Touch Experiences and Attitudes Questionnaire (TEAQ) is a widely applicable self-report tool. The purpose of our research was to examine the validity and reliability of the Chinese version of TEAQ for Chinese college students. We translated the 57 items of the original TEAQ into Chinese and assessed its cultural and linguistic adaptation in Chinese context. Two samples were recruited for the assessment of validity and reliability. The Social Support Rating Scale, Index of Well-being Scale and Security Questionnaire were chosen as criterion-related validity indicators. Item analysis, principal component analysis and confirmatory factor analysis indicated that the simplified Chinese version of TEAQ contained 18 items in three factors: Attitude to Intimate Touch, Childhood Touch and Current Positive Touch, with a cumulative variance contribution rate of 57.12%. The 3-factor model had good validity and reliability. The TEAQ was positively correlated with social support, sense of security and well-being. There were demographic differences in sex and left-behind experience. We anticipate the simplified TEAQ will be a valuable tool for the research of touch among Chinese college students.

## Introduction

Social touch plays an important role in social communications. Individuals intentionally touch others’ bodies to convey emotional messages in interpersonal interactions, including touching, holding hands, hugging, snuggling, patting and other behaviors [1]. The expanded Social-Affective Touch Hypothesis proposes that C-tactile stroking and deep pressure are two primary sensory inputs for pleasant, rewarding social touch [2]. C-tactile afferents in hairy skin constitute a privileged peripheral pathway for pleasant tactile stimulation that is likely to be social touch [3]. Moderate deep pressure which occurred in hugs, cuddling, and massage may constitute another important form of social touch [2]. Both of them have similar affective effects, including similar ratings of touch pleasantness and increased ratings of calm, and can activate highly similar brain regions.

Social touch is central to an individual’s early interpersonal interactions and physical self, promoting a balance between internal physiological states and responses to external stimuli [4]. It is both a sensory tool for people to explore the world and induces positive emotions, and is closely related to physical and mental health [5]. Touch helps create and strengthen interpersonal affective connections [6] and plays an important role in interpersonal interactions [7]. The frequency and duration of a mother’s touch on her infant can predict the development of infant’s relevant brain regions responsible for interpersonal functions [8], and can also influence the development of social emotions and the quality of parent-child attachment [9]. Touch between romantic partners enhances psychological intimacy and heightens affective bonds. Partners who were touched more can maintain higher levels of psychological well-being both currently and 6 months later [10, 11]. Social touch can also help individuals better cope with stress and maintain psychological well-being. For example, when an individual suffers pain from thermal stimulation, being touched by a partner is effective in reducing the subjective level of pain [12]. After receiving another person’s touch, individuals are able to diminish negative evaluations and enhance positive evaluations [13]. Individuals in the aversive condition show a higher subjective need for the touch of others [14]. Touch is also able to be used clinically, where doctors’ touch on patients can improve the quality of interpersonal communication between doctors and patients [15].

Research in the field of touch has only recently begun in China, but in recent years, the number of related studies has steadily increased, and it is expected that this area will get a lot of attention from researchers in the future [16]. Few assessment tools with the factor structure has been determined and validated to measure social touch in Chinese culture. Only Bai et al. introduced the Nurse’s Comfort with Touch Scale (NCTS) which can measure the nurses’ comfort level when touching patients in clinical work [17]. This questionnaire can only be used clinically and has a narrow applicability. It cannot evaluate the touch experiences and attitudes in daily life. The Touch Experiences and Attitudes Questionnaire (TEAQ) which was developed by Trotter et al. measures individuals’ current and childhood experiences of positive touch, as well as attitudes to positive touch [18]. The TEAQ is applicable to early adult population, and is now a more widely applicable self-report assessment tool. Participants were mainly from college students, 60% of whom had an intimate partner. In order to ensure applicability, the questionnaire use terms "someone they like, someone they are close to" instead of "boy/girlfriend, partner". The TEAQ consists of 57 items in 6 factors: Current Intimate Touch (CIT), Attitude to Intimate Touch (AIT), Friends and Family Touch (FFT), Attitude to Unfamiliar Touch (AUT), Attitude to Self-Care (ASC) and Childhood Touch (ChT). CIT, AIT, FFT, AUT and ASC measure individuals’ current experiences and attitudes to various types of touch at present. They can be used to reveal individual differences in experiences of and attitudes to positive touch, and to help researchers to conduct studies on touch. ChT can measure a person’s early experience of social touch. Previous research suggested that Childhood Touch can be used separately to explore the impact of childhood touch deprivation on adult mental health in special groups such as care leavers and people with left-behind experience [19]. Left-behind experience means that an individual remained at home when one or both of his/her parents worked in urban areas for at least six months before the age of 18 [20]. The “touch” in the questionnaire refers to all types of touching that occurs during interpersonal interactions, as well as non-interpersonal touching behaviors (e.g., using bath essence when having a bath) and does not involve linear touching behaviors performed with soft brushes in experimental studies. This means that the ecological validity of TEAQ is relatively higher. In view of these advantages, the TEAQ has been adopted by researchers in several countries, including the UK and the Netherlands, since its introduction [19, 21]. It has been introduced into multiple versions in Russian, Portuguese and Mongolian [22–24].

Touch is a powerful tool for communicating in different cultures. Suvilehto et al. found that individuals in both East Asian and Western cultures can establish affective bonds through touch, and different cultures have the same touch taboos, with people allowing closer people to touch body parts more often than strangers [25]. However, there are still significant differences in touch cultures and expressions in different regions. China belongs to a low touch cultural-level, and the touch prevalence and touch diversity among Chinese people are far below the world average [26]. In particular, a certain physical distance should be maintained between men and women. China had a tradition in the past: even for those who are in a romantic relationship, it was not appropriate to touch each other in public. It can be inferred that the potential of social touch among strangers in China may be lower.

Cultural differences in social touch lead to natural differences in the number of items and factor structures of different national versions of TEAQ. For example, the Russian version of TEAQ was reduced to a 37-item questionnaire characterized by five-factor structure [24]. In the present research we aimed to validate a Chinese version of TEAQ. Because there are few tools to measure the experiences or attitudes towards social touch in Chinese population, we would first verify the applicability of TEAQ in college students. The present research was conducted in two studies: exploring the component structure of the Chinese version of TEAQ (study 1); a reliability and validity test of the version obtained in study 1 and 2.

## Study 1

The purpose of study 1 was to assess the cultural and linguistic adaptation of 57 items of the original TEAQ for Chinese culture. A subset of items with sufficient cultural appropriateness was selected. The component structure of this new version of TEAQ was then determined.

### 1.1 Methods

#### 1.1.1 Participants

A total of 499 college students was recruited via mental health course and were compensated for credit in September 2022, 211 (42.3%) were male and 288 (57.7%) were female. Their age was 18.25 ± 0.90 years. Inclusion criteria included being college students, and completing all questionnaires. From an initial sample of 523, 24 participants were excluded for repeatedly selecting the same option. This research was approved by the Ethics Committee of Xinxiang Medical University. All subjects gave their written informed consent and anonymously completed all questionnaires.

#### 1.1.2 the Chinese version of TEAQ

Two graduate students majoring in psychology translated independently the English version into Chinese, and then compared the two versions and reached a consensus on the translated items. Then, 2 graduate students majoring in English who majored in double majors in psychology at the undergraduate level back-translated independently the translated items and then reached a consensus. They compared the back-translated manuscript with the English version, and amended any ambiguities, such as changing the original item 45 " I often have my skin stroked " to "I often get touched by others". Finally, two psychology teachers with rich experience in translating English books conducted a check with the English items, translations and back-translations to ensure the consistency of item translations. We also consulted the original author to comment upon the back-translations.

Then 40 college students were randomly recruited to examine the content validity (22 females, 18 males; *M*age = 20.95 ± 2.47). They were asked to assess the clarity, understanding, cultural relevance of the translations, and supplement positive touch experiences not included in the 57 items. We based on two criteria to revise it: (1) Items deemed inappropriate by at least 20% of the participants were to be excluded [24]. (2) Positive touch with others in an appropriate social environment proposed by at least 20% of the participants were to be added.

The items related to kissing and hugging were excluded, for they did not meet the Chinese greeting and parting etiquette (e.g., “I find it natural to greet my friends and family with a kiss on the cheek”). Participants reported that having sex and other similar behaviors are very private, and should not fall under the category of social touch in daily life. In addition, college students’ sexual behavior is often known as premarital sexual behavior which has long been disapproved in China [27]. The incidence of sexual intercourse among Chinese college students is relatively low. So items related to sex were excluded. Total 16 items were excluded, including item 4, 10, 12, 14, 15, 16, 19, 26, 27, 30, 36, 41, 44, 46, 48, and 49.

Participants also reported that they often received kiss and pat from their parents during childhood. So we added 2 items: “As a child my parents would often kiss me”; “As a child my parents would often pat my shoulders or head”. Finally, 43 items were selected for next analysis. These items were reordered from 1 to 43, using a 5-point Likert scale as the English version (please see S1 File).

#### 1.1.3 Statistical analysis

To examine the suitability for conducting a confirmatory factor analysis (CFA), we first tested the normality of the data, Kaiser-Meyer-Olkin test and Bartlett’s sphericity test using SPSS 24.0. Then confirmatory factor analysis (CFA) was conducted to assess the 6-factor model using Mplus 7.0. The following indexes and cut-offs were considered to determine the goodness of model fit: χ^2^/df ≤ 5 acceptable fit; CFI values ≥ 0.90 acceptable fit; TLI values ≥ 0.90 acceptable fit; and RMSEA values ≤ .08 good fit [28, 29]. When the model did not fit the data well, item analysis and principal component analysis (PCA) were carried out by using SPSS 24.0.

### 1.2 Results

The results of normality analysis showed no significant biases in relation to the averages, with values lower than |3| and |10| for skewness and kurtosis, respectively [30]. The Kaiser-Meyer-Olkin test and Bartlett’s test showed that KMO = 0.91, *p* < 0.001, above the recommended criterion of 0.60. Results of the CFA demonstrated that the 6 factor model did not fit our data well (χ^2^/df = 3.255, CFI = 0.763, TLI = 0.746, RMSEA = 0.067). Following poor model fit indices, we conducted item analysis and PCA [31, 32].

#### 1.2.1 Item analysis

According to the Russian TEAQ, the total score of 43 items were ranked, and the top 27% and the bottom 27% were respectively set as high and low groups [24]. The independent samples *t* tests were conducted on the scores of each item. The results showed that there were no significant differences between the high and low groups of items 3 and 26. The differences were significantly in the remaining items (*p* < 0.001). Items 3 and 26 were excluded from further analyses. Then Pearson correlations were performed between each item and total score. Item-total correlations revealed that 9 items (Items 1, 2, 3, 4, 16, 23, 26, 29, 38) had item-total correlations below 0.30 and should be excluded [33].

#### 1.2.2 Principal component analysis

We conducted PCA on the remaining 34 items. The Kaiser-Meyer-Olkin test and Bartlett’s test showed that KMO = 0.92, *p* < 0.001, indicating that the dataset was suitable for PCA. PCA with varimax rotation was performed to extract the component structure [24]. The following criteria served as exclusion criteria [24, 31]. (1) The item communality was less than 0.30. (2) Item loading was less than 0.4. (3) Item loadings on two components were all greater than 0.4, and the difference of loading was less than 0.2. (4) The number of items contained in one components was less than 2 or equal to 2. If any one of the criteria was met, the corresponding item was excluded. Finally, a total of 16 items were excluded. A three-component structure was extracted from the 18 retained items, with a cumulative variance contribution rate of 57.21% (Table 1). Component 1 was “Attitude to Intimate Touch” (AIT), including 8 items, which related to attitudes to touch with intimate partners. Component 2 was “Childhood Touch” (ChT, 6 items), which related to the positive touch experiences that individuals experienced early in their development. Component 3 was “Current Positive Touch” (CPT), which combined two components of the original TEAQ (“Friends and Family Touch” and “Current Intimate Touch”). Component 3 included 4 items, which related to interpersonal physical touch experiences with intimate partner, family members and friends. Two components presented in the English version were excluded from the Chinese version. One was Attitude to Unfamiliar Touch, the other was Attitude to Self-Care.

**Table 1:** The componet structure of Chinese version.

Items of the simplified Chinese version, with numbers		AIT	ChT	CPT
32. I like to stroke the skin of someone I know intimately		0.768		
21. I enjoy the feeling of my skin against someone else’s if I know them intimately		0.752		
15. I enjoy having my skin stroked		0.745		
33. Snuggling up on the sofa with someone is great		0.739		
20. I enjoy being cuddled by someone I am fond of		0.711		
28. I enjoy holding hands with someone I am fond of		0.690		
12. It feels really good when someone I am fond of runs their fingers through my hair		0.674		
18. I can always find somebody to physically comfort me when I am upset		0.555		
19. As a child my parents would often kiss me			0.816	
42. There was a lot of physical affection during my childhood			0.709	
22. As a child my parents would tuck me up in bed every night and give me a hug and a kiss goodnight			0.701	
24. As a child my parents would often pat my shoulders or head			0.675	
6. As a child I would often hug family members			0.622	
9. As a child my parents would often hold my hand when I was walking along with them			0.510	
43. I like to link arms with my friends and family as I walk along				0.791
10. I often hold hands with someone I know intimately				0.705
25. I often make physical contact with my friends and family when I am with them				0.610
41. I like it when my friends and family greet me by giving me a hug				0.602
Total eigenvalue	10.3	7.03	2.19	1.08
Percentage of variance explained	57.21%	24.71%	17.58%	14.92%

***Note***. Blanks indicate where component loadings are below 0.4. Component 1: Attitude to Intimate Touch (AIT); Component 2: Childhood Touch (ChT); Component 3: Current Positive Touch (CPT). The 18 items expressed in Chinese were given in S2 File.

## Study 2

The purpose of study 2 was to examine the construct and criteria validity and reliability of 18-item Chinese version. We replicated the three-factor structure of revised TEAQ. Social baseline theory explains that close proximity to social resources is the baseline assumption of the human brain, and health and well-being can be improved by access to close social relationships [34]. Social touch is one of the important ways of interpersonal communication, and can provide more support than the presence of others [35]. Researches indicated that social touch can provide strong support signals and increase one’s well-being and the sense of security [36, 37]. Four factors in the original TEAQ, including Attitudes towards Intimate Touch, Childhood Touch, Current Intimate Touch, and Friends and Family Touch, all had significant positive correlations with social support [18]. Social touch can deliver love and improve individuals’ sense of comfort. Early maternal touch affects peoples’ well-being in childhood [38]. Social touch can also help partners defuse conflict and enhance their relational well-being [37]. A sense of security was a feeling that can face and accept the possible future risk (physical or mental), often expressing as a sense of certainty and control [39]. Social touch plays a protective factor for individuals, and increase the sense of security. Researches revealed that social touch can help people ignore threatening information and even lead them to increase risk taking behavior. Harlow’s famous study found that the touch of a baby monkey to the flannel mother monkey could enhance the sense of security of the baby monkey [40]. In the present study, we chose social support, well-being and sense of security as criterion-related validity indicators.

The perception of touch can be affected by factors related to social and cultural context [18]. Previous researches have consistently shown sex differences in touch experiences and attitudes. Girls receive more parental touch than boys during their childhood [23]. For adults, females tend to use social touch more frequently in interpersonal interactions, and feel more comfortable with social touch [41, 42]. The perception of touch can also be affected by early touch experience. Individuals who rarely experienced touch evaluated the pleasantness of touch lower than those who experienced touch often [19]. We will evaluate the sex effects and experience effects on the simplified TEAQ responses.

### 2.1 Methods

#### 2.1.1 Participants

A total of 709 college students was randomly recruited in November 2022, 285 (40.2%) were male and 424 (59.8%) were female; 135 (19.04%) were students with experience of left-behind and 574 (81.96%) were students without experience of left-behind. The average age was 19.11 ± 1.50. Inclusion criteria included being college students, and completing all questionnaires. From an initial sample of 805, 57 participants were excluded for repeatedly selecting the same option, and 39 for not completing all questionnaires. All students gave their written informed consent and voluntarily agreed to answer the questionnaires.

#### 2.1.2 Measures

*The 18-item TEAQ*. Participants were asked to completed the simplified version of TEAQ from study 1.

*Social Support Rating Scale (SSRS)*. The SSRS consists of 10 items, rated on a four-point scale [43]. The scale is divided into three dimensions: objective support, subjective support and utilization of support. The word “colleague” and “workplace” in items was changed to “classmate” and “school”. The higher score indicates higher level of social support. The SSRS demonstrated good test-retest reliability (intraclass correlation coefficient was 0.92). In the present study, Cronbach’s *α* was 0.713.

*Index of Well-being Scale (IWB)*. The IWB is a 7-point scale with nine items and two subscales, namely the index of general affect and the index of life satisfaction [44]. All items were reverse-scored, with higher score representing a higher sense of well-being. The Chinese version demonstrated good test-retest reliability (intraclass correlation coefficient was 0.82) [45]. In our study Cronbach’s α was 0.940.

*Security Questionnaire (SQ)*. The SQ consists of 16 items and is divided into two dimensions: interpersonal security and certainty in control [46]. The word “leader” in question 10 was changed to “teacher” [47]. The scale was rated on a 5-point scale, with the higher the score, the higher the sense of security. The SQ demonstrated good internal consistency (Cronbach’s α = 0.80). In this sample, Cronbach’s α was 0.91.

#### 2.1.3 Statistical analysis

We used Mplus 7.0 for CFA, and used SPSS 24.0 for reliability and criterion validity testing. Reliability was assessed through Cronbach’s alpha values (*α*≥0.70 means considered satisfactory) [48]. Criterion validity was assessed though Pearson’s correlation coefficients between TEAQ and SSRS, IWB and SQ (the correlation coefficients of 0.12, 0.24, and 0.41 are interpreted as the cut-offs for small, medium, and large effects, respectively) [49]. Independent samples *t* test and effect size were calculated to clarify the meaningfulness of demographic differences (Cohen’s *d*: 0.2-small effect size; 0.5-medium effect size; 0.8-large effect size) [50, 51].

### 2.2 Results

#### 2.2.1 Confirmatory factor analysis

We carried out a CFA analysis including a total score to develop a higher-order model where each item loaded for only one factor. The results demonstrated that χ^2^/df = 6.028, CFI = 0.900, TLI = 0.884, RMSEA = 0.084. A modified Model 2 considering covariance of errors for items with similar content (item pairs Q12-Q15, Q18-Q20, Q20-Q21, Q28-Q18, Q32-Q33, Q22-Q24, Q10-Q43) showed more satisfactory fit (χ^2^/df = 3.880, CFI = 0.946, TLI = 0.933, RMSEA = 0.064). The modified three-factor model revealed a good fit and had good structural validity.

#### 2.2.2 Reliability

The total Cronbach’s *α* was 0.917. The split-half reliability coefficient of the questionnaire was 0.895.

#### 2.2.3 Criteria validity

We averaged the scores for AIT, ChT and CPT and then summed these three average scores as the total TEAQ score. Pearson’s correlation showed that the simplified TEAQ and three subscales were significantly positively correlated with social support, sense of security and well-being (*r* = 0.10 ~ 0.37, *p* < 0.01) (Table 2).

**Table 2:** Correlations of TEAQ with social support, well-being and sense of security.

	TEAQ	AIT	ChT	CPT
Social support	0.36**	0.23**	0.31**	0.37**
Well-being	0.25**	0.10*	0.28**	0.24**
Sense of security	0.20**	0.12**	0.21**	0.19**

*Note*.

* *p* <0.05,

** *p* <0.01

#### 2.2.4 Demographic differences

We tested the differences of TEAQ response in demographic variables using independent samples *t* tests (Table 3). The results showed TEAQ score and subscale scores for participants with left-behind experiences were significantly lower than those without left-behind experiences (*p* < 0.01, Cohen’s *d = 0*.*25 ~ 0*.*42*). However, 4 items out of 6 in Cht factor refer to parental touch, which may not be suitable for students with left-behind experiences. We calculated the average score of items 6 and 42 which did not refer to parental touch as another indicator, and the difference was also significantly (*p* < 0.01, Cohen’s *d* = 0.48). We also found sex differences. The scores for females were significantly higher than those for males (*p* < 0.01, Cohen’s *d* = 0.26 ~ 0.92).

**Table 3:** Descriptive statistics of demographic variables.

	TEAQ	AIT	CPT	ChT	ChT (6, 42)
Non leftbehind (*n* = 574)	3.34 ± 0.78	3.52 ± 0.85	3.30 ± 0.98	3.21 ± 0.93	3.19 ± 1.02
leftbehind (*n* = 135)	3.00 ± 0.82	3.30 ± 0.94	2.91 ± 1.04	2.78 ± 0.95	2.69 ± 1.08
*t*	4.56***	2.65**	4.10***	4.73***	5.03***
Cohen’s *d*	0.42	0.25	0.39	0.46	0.48
Females(*n* = 424)	3.47 ± 0.73	3.63 ± 0.77	3.57 ± 0.89	3.22 ± 0.95	
Males (*n* = 285)	2.98 ± 0.80	3.25 ± 0.96	2.72 ± 0.95	2.98 ± 0.93	
*t*	8.41***	5.83***	12.12***	3.42**	
Cohen’s *d*	0.64	0.44	0.92	0.26	

*Note*.

** *p* <0.01,

*** *p*<0.001

## 3 Discussion

Currently, there is a lack of questionnaires in China that measure social touch experiences and attitudes to young adults. The TEAQ is a widely applied self-report assessment tool that has been introduced into multiple versions. The purpose of our research was to introduce the original TEAQ into Chinese. The simplified Chinese TEAQ contained 18 items and has 3 factors. The simplified questionnaire had good validity and reliability in Chinese college students. The 3-factor model had good construct validity. The Cronbach *α* coefficient and split-half reliability of the simplified TEAQ all met the measurement requirements, and were close to the Cronbach *α* coefficient (0.81–0.93) of the 6 factors of the original questionnaire. There were significantly positive correlations between TEAQ and social support, well-being and sense of security.

### 3.1 The simplified Chinese version of TEAQ

Compared with the original TEAQ, the simplified Chinese version was characterized by a clear three-factor structure (Attitude to Intimate Touch (AIT), Childhood Touch (ChT), and Current Positive Touch (CPT). Although China belongs to a low-touch culture, there is no significant difference between Chinese and Western subjects in the experience of touch pleasure, which shows that the differences between China and Western high-touch culture countries are mainly reflected in the culture of how to use touch in social interaction norms rather than personal pleasure in touch [52]. Since Chinese people also get a high sense of pleasure from social touch with acquaintances, they hold a positive attitude towards touch. Therefore, the Chinese version of TEAQ still retains the factor of AIT, which has the largest number of items and the highest contribution rate (24.71%). The 3-factor model also included individuals’ early touch experiences. Touch is one of the important ways of parent-child communication. Kangaroo mother care which provides contact between mother’s chest skin and infants’ skin is an effective intervention to reduce the rate of premature infants in mortality and complication. China is vigorously promoting kangaroo mother care in clinical practice [53]. The touch experience in early development plays an important role in the healthy development of children [8]. Individuals with a lower early touch experience have lower evaluation of the pleasure of touch behavior in adulthood [54]. So the simplified version should include ChT factor which explains 17.58% of the variable. Items of factor CPT are derived from Friends and Family Touch (FFT) and Current Intimate Touch (CIT) in the original questionnaire. There may be two possible explanations. Firstly, due to poor cultural adaptability, these questions have been excluded (Please see the next paragraph for a detailed explanation). Secondly, cross-cultural research also found that the incidence and diversity of touch behaviors between Chinese people and relatives and friends are far lower than the world level [26]. That is, touch behaviors rarely occur in daily life. Therefore, FFT and CIT presented in the original questionnaire are combined into one factor (CPT) which has only 4 items.

Two factors (Attitude to Self-Care (ASC) and Attitude to Unfamiliar Touch (AUT)) were not presented in the simplified version. As for the Chinese, the main purpose of various skin care and grooming behaviors (e.g. taking a bath or using shower gel) is cleanliness and beauty, and few people consider the impact on individual pleasure. Trotter et al. also found that the ASC had a lower correlation coefficient with the other five factors [18]. In addition, the fact that females comprised 70% to 80% of the total number of participants in previous researches may also be a reason [18, 23]. Females pay more attention to their appearance and self-care [55]. So factor ASC was excluded in the simplified TEAQ. As for factor AUT, the degree of intimacy in interpersonal relationships is different, and the spatial distance between the two parties is also different. Chinese touch prevalence towards partners and friends is already at an extremely low level in 45 countries [26]. It can be inferred that positive touch rarely occurs between individuals and unfamiliar people in China. The Attitude to Unfamiliar Touch factor was also not reproduced on the Russian version of TEAQ [24].

What needs to be discussed specifically is that only 18 items were retained. On the surface, this seems to be problematic. However, we have the following reasons to believe that the 3-factor model is simplified and scientific. On the one hand, the original TEAQ involves 6 different aspects of touch, from childhood touch to current touch and from interpersonal touch to self-touch. Compared with other social touch questionnaires, TEAQ is characterized by a large number of items [56]. Although 6 factors explained 56% of the variance, the percentage of explained variance decreased steeply after the second factor, such as 5.6%, 4.1%, and 3.3% for ASC, AIT and AUT factors, respectively. A solution with no more than 3 factors may result in a relatively parsimonious structure. In the simplified TEAQ, factors ASC and AUT are excluded, and AIT is merged into CTP. The percentage of variance explained by the retained 3 factors slowly decreased (24.71%, 17.58% and 14.92%). On the other hand, these items were excluded from the simplified model for three reasons. Firstly, the kissing, hugging and sex-related items were excluded in “cultural and linguistic adaptation” section, which is consistent with previous researches. Researches on the positive effects of touch mainly focus on the nonsexual touch [52]. Sex-related items were also excluded in revision of Russian TEAQ [24]. In addition, a total of 3 items were excluded from the Chinese version of NCTS, such as “Bathing the patient” and “Washing the patient’s genital area” [17]. These items are related to the nurses’ operations in clinical work. However, both nurses and patients may feel uncomfortable and awkward influenced by Chinese social culture. Secondly, as for the social touch behavior between acquaintances, the prevalence and diversity of touching not only a female/male friend and one’s own child, even that of touching one’s romantic partner were far below the world average level [26, 57]. Finally, as in the Russian version, we used the total score in the revision of TEAQ [24]. The use of total score may have the potential to enhance the associations between each items. Those items which are less relevant to positive touch experiences and attitudes are likely to be excluded by using a total score, such as factor ASC and AUT.

Too many items may make TEAQ not easy to promote and use. Trotter, the author of TEAQ, suggested that future research could consider creating a shortened version. The Russian TEAQ remains 37 items and 5 factors. Our research may implement her prospect: a simplified Chinese version may be more convenient for promotion and for use. In short, there is a big difference between the simplified 3-factor model and the original questionnaire, which verifies that there are both commonalities and differences in touch cultures and expressions in different regions. From another perspective, this research can also verify a contrast that although Chinese people enjoy the pleasure of social touch, the prevalence and diversity of touch behaviors are very low. Future research could explore how to spread the touch culture in China to increase the prevalence and diversity of touch behavior.

### 3.2 The association between social touch and criterion variables

Correlation analysis showed that the scores of simplified TEAQ and 3 factors were significantly positively correlated with those of social support, well-being and the sense of security. This is consistent with previous researches and validates the criterion validity of the simplified version. In line with the results of original TEAQ, there is a positive relationship between social support and touch experiences and attitudes [18]. Schirmer et al. put forward the tool theory of touch, believing that touch is an important tool for interpersonal communication [58]. The initiator of touch can pass on emotions such as love and social support to the touched person through touch behavior, which can effectively provide social support for individuals [59]. Individuals are more inclined to hug their partners when they want to be comforted [1]. Social touch has an effect of social support, which can effectively alleviate the individual’s feelings and coping with pain [60].

The touch experiences and attitudes were found to be associated with well-being. Social touch can deliver love and improve individuals’ sense of comfort. Early maternal touch affects people’s well-being in childhood [38]. Jakubiak and Feeney proposed a theoretical model that depicts a causal relationship between receiving social touch in adults’ close relationships and long-term beneficial outcomes [61]. Social touch could also affect well-being by reducing stress. For example, engaging in touch prior to and during conflict was effective in improving conflict behaviors of couples and to buffer stress in real and imagined contexts [37]. So if individuals receive more parental touch during their childhood and have a more positive experience and attitude to social touch in adulthood, they also tend to experience higher levels of well-being.

Regarding the sense of security, Harlow found that the touch of a baby monkey to the flannel mother monkey can enhance the sense of security of baby monkey [40]. Another study found that the parent’s touch to the child can significantly reduce the children’s alertness to threatening stimuli [62]. The toucher will convey a feeling of intimacy and caring to the touched person, and the touched person will feel the emotional connection and gain a sense of security [10, 37]. Although the correlation coefficients between scores of TEAQ and sense of security in our research were relatively low, it also revealed that individuals with higher touch experiences and attitudes are accompanied by higher sense of security.

### 3.3 Demographic differences

There were demographic differences in the simplified TEAQ. The results revealed that TEAQ scores of males were significantly lower than that of females, which was consistent with previous research. Both of the English and Russian versions of TEAQ indicated a consistent sex effect [24]. In a romantic relationship, female is the strongest predictor of overall desire for touch [37]. A cross-cultural research found that female had a greater preference for touch and felt more comfortable, whether the participants were from Hong Kong or Germany [63]. In summary, the fact that females have a better experience of and attitude to touch than males is culturally universal.

We also found that individuals with left-behind experience got lower TEAQ scores than those without left-behind experience. Currently, there is little research exploring the impact of left-behind experience on individual touch experiences and attitudes. However, some studies have explored the impact of early touch loss. Sailer and Ackerley found that continued exposure to touch affected experience of touch [54]. Individuals with self-reported low touch exposure rated the slow stroking and touch from familiar persons as less pleasant. Care leavers who have spent a minimum of 1 year in foster care reported significantly lower levels of positive childhood touch compared to non-care leavers [19]. It can be inferred that left-behind children spend less time with their parents and have less chance to be touched by their parents and other caregivers. After attained adulthood, they were very likely to report lower level of childhood touch, and the left-behind experience may alter their current touch experiences and attitudes.

## Conclusions

The simplified Chinese version of TEAQ contains 18 items, and has distinct and reliable 3-factor structure: Factor 1—Attitude to Intimate Touch; Factor 2—Attitude to Intimate Touch and factor 3—Current Positive Touch. An average score for each subscale needs to be calculated first and then summed as the total TEAQ score. The comparison between the 3-factor model and the original 6-factor model indicates significant differences in touch experiences and attitudes among different cultures. The Chinese version has good validity and reliability in Chinese college students. We anticipate this questionnaire will become a valuable tool for investigating the association between social touch and physical and mental health. We also look forward to continuously increasing the prevalence and diversity of touch behavior and promoting touch culture in China through future research.

## Limitations

At present, there is no available questionnaire with the factor structure has been determined and validated in China. People from different age groups may have different touch experiences, so our research only selected college students as samples. The participants in our research were conservatively taken from college students. This may limit the promotion of the Chinese version. Future studies should continue to explore the applicability of TEAQ on more diverse samples. Additionally, there have been some special groups of children in China, such as left-behind children. They have little chance of being touched by their parents. But most items of Cht factor refer to parental touch, which maybe a limitation of the TEAQ. In order to make the TEAQ more accurate and relevant to a wider range of participants, the term “parental touch” should be revised to “caregivers’ touch”.

## Supporting information

S1 File The 43-item version, reordered. (DOCX)

S2 File The simplified TEAQ in Chinese. (DOCX)
