# Supplementary material for: Reliability and validity of a simplified touch experiences and attitudes questionnaire for Chinese college students
Source: PLoS One. 2024 Jan 2;19(1):e0295812. doi: 10.1371/journal.pone.0295812 (PMC10760887; doi:10.1371/journal.pone.0295812)
Supplement: S1 File — (DOCX) [file pone.0295812.s001.docx]

S1. the 43-item Version, Reordered

1. I dislike people being very physically affectionate towards me

2. I like using body lotions

3. I have to know someone quite well to enjoy a hug from them

4. I like to use bath essence when having a bath

5.I find stroking the hair of a person I am fond of very pleasurable

6. As a child I would often hug family members

7. I often snuggle up on the sofa with someone

8. I like to link arms with my friends and family as I walk along

9. As a child my parents would often hold my hand when I was walking along with them

10. I often hold hands with someone I know intimately

11. When I am upset, there is usually someone who can comfort me

12. It feels really good when someone I am fond of runs their fingers through my hair

13. I regularly hug people I am close to

14. My life lacks physical affection

15. I enjoy having my skin stroked

16.I often take a shower or bath with someone

17.I am put off by physical familiarity

18.I can always find somebody to physically comfort me when I am upset

19. As a child my parents would often kiss me

20. I enjoy being cuddled by someone I am fond of

21.I enjoy the feeling of my skin against someone else’s if I know them intimately

22.As a child my parents would tuck me up in bed every night and give me a hug and a kiss goodnight

23. If someone I don’t know very well puts a friendly hand on my arm it makes me feel uncomfortable

24. As a child my parents would often pat my shoulders or head

25. I often make physical contact with my friends and family when I am with them

26. It makes me feel uncomfortable if someone I don’t know very well touches me in a friendly manner

27. As a child my mother regularly brushed my hair

28. I enjoy holding hands with someone I am fond of

29. I like exfoliating my skin

30. I often get touched by others

31. As a child my parents always comforted me when I was upset

32. I like to stroke the skin of someone I know intimately

33. Snuggling up on the sofa with someone is great

34. I often put my arm around a close friend as we walk along together

35. My mother regularly bathed me as a child

36. I like having a bath with lots of bubble bath

37. I don’t get many hugs these days

38. I am often given a shoulder massage

39. I like to use face masks on my skin

40. My parents were not very physically affectionate towards me during my childhood

41. I like it when my friends and family greet me by giving me a hug

42. There was a lot of physical affection during my childhood

43. I often link arms with my friends and family as I walk along
