# Supplementary material for: Reliability and validity of a simplified touch experiences and attitudes questionnaire for Chinese college students
Source: PLoS One. 2024 Jan 2;19(1):e0295812. doi: 10.1371/journal.pone.0295812 (PMC10760887; doi:10.1371/journal.pone.0295812)
Supplement: S2 File — (DOCX) [file pone.0295812.s002.docx]

**S2. 中文简版TEAQ**

6.小时候我经常拥抱家长。

9.小时候跟家长一起走路时，他们经常拉着我的手。

10.我经常和亲密的人拉手。

12. 所喜爱的人的手指在我发丝间游走的感觉非常美好。

15.我喜欢肌肤被抚摸的感觉。

18. 当心情不好时，总是有人以身体接触的方式安慰我。

19.小时候家长经常亲我。

20.我喜欢被喜爱的人拥抱。

21.我喜欢和亲密的人身体接触。

22.小时候临睡前，家长都会轻轻拍着哄我睡觉，或者给我一个拥抱和吻。

24.小时候家长经常拍拍我的头或肩膀。

25.和朋友或家人在一起的时候，我经常和他们有身体接触。

28.我很享受和喜爱的人牵手的感觉。

32.我喜欢抚摸亲密的人。

33.和他人依偎在一起的感觉很美好。

41.我喜欢朋友和家人用拥抱来迎接我。

42.小时候家长经常以身体接触的方式来表达对我的爱。

43.我经常和朋友或家人挽着胳膊散步。

注：计分方法为先计算3个因子的平均分，然后相加作为总分。
